# Histopathology of Sinonasal and Nasopharyngeal Neoplastic Lesions in a Tertiary Care Center of Western Nepal: A Descriptive Cross-sectional Study

**DOI:** 10.31729/jnma.6859

**Published:** 2021-07-31

**Authors:** Sudeep Regmi, Arnab Ghosh, Dilasma Gharti Magar, Sushma Thapa, Krishna Prasad Koirala, Om Prakash Talwar

**Affiliations:** 1 Department of Pathology, Manipal Teaching Hospital, Phulbari, Pokhara, Nepal; 2 Department of ENT-Head and Neck Surgery, Manipal Teaching Hospital, Phulbari, Pokhara, Nepal

**Keywords:** *histopathology*, *nasal cavity*, *nasal polyp*, *squamous cell carcinoma*

## Abstract

**Introduction:**

Sinonasal and nasopharyngeal regions harbor diverse clinical conditions including many non-neoplastic and neoplastic lesions presenting with nasal obstruction, facial pain and swelling, rhinorrhea, epistaxis, and other oral, ear, and orbital symptoms. Histopathology of excised lesions is the mainstay for the definitive diagnosis and management of neoplastic lesions. The aim of this study is to find out the prevalence of neoplastic lesions among sinonasal and nasopharyngeal masses.

**Methods:**

This was a descriptive cross-sectional study conducted among sinonasal and nasopharyngeal masses in the department of pathology of a tertiary care center of western Nepal with primary data of 20 years from January 2001 to May 2020. Ethical approval was taken from the Institutional Review Committee. Convenience sampling method was used. Data management and statistical analysis were done using Statistical Package for Social Sciences. Point estimate at 95% Confidence Interval was calculated along with frequency and percentage.

**Results:**

Out of 395 sinonasal and nasopharyngeal lesions, 134 (33.92%) (29.26-38.58 at 95% Confidence Interval) were neoplastic lesions. The malignant lesions were found to be 60 (44.77%). Inverted papilloma was the most common benign lesion comprising 28 (20.89%) of cases, and squamous cell carcinoma and nasopharyngeal carcinoma were the most common malignant lesions comprising 12 (8.95%) cases each.

**Conclusions:**

This study observed a variety of neoplastic lesions. The most common benign lesion was nasal polyp and squamous cell carcinoma and nasopharyngeal carcinoma were the most common malignant lesions.

## INTRODUCTION

Sinonasal (SN) and nasopharyngeal (NAP) region harbors diverse clinical conditions ranging from different nonneoplastic to neoplastic lesions.^1-4^ Many of these lesions present as polypoidal masses with nasal obstruction, whereas other presenting symptoms may be facial swelling and pain, rhinorrhea, and epistaxis along with associated oral, ear and orbital symptoms.^5-8^

A presumptive diagnosis can be given on the basis of clinical symptoms and imaging techniques, but histopathology examination (HPE) is essential to confirm the definitive diagnosis. HPE can be taken as the gold standard to diagnose and categorize neoplastic lesions of the SN and NAP region.^5,7^

The aim of the study is to find out the prevalence of neoplastic lesions among sinonasal and nasopharyngeal masses.

## METHODS

This was a descriptive cross-sectional study conducted among patients with sinonasal and nasopharyngeal masses in the department of pathology, Manipal Teaching Hospital, Phulbari, Pokhara, Nepal with primary data of 20 years from January, 2001 to May, 2020. Ethical approval was obtained from the Institutional Review Committee prior to commencement of the study (MEMG/IRC/358/GA). Convenience sampling method was used and sample size was calculated using the formula,

Sample size calculation

n = Z^2^ × p × q / e^2^

  = 1.96^2^ × 0.5 × (1-0.5) / (0.05)^2^

  = 384

Where,

n = sample size
Z = 1.96 at 95% Confidence Interval
p = prevalence at 50% for maximum sample size
q = 1-p
e = margin of error, 5%

The sample size 395 were taken for the study. Data was retrieved from the departmental archive and all the cases with histopathological evaluation of SN a nd NAP masses were involved in the study. Out of 1849 otolaryngorhinology cases referred to Pathology Department for HPE, 395 cases were of SN and NAP masses in last 20 years duration. About 37 cases presented with recurrence of the lesion. The study was recorded in Microsoft Excel with the help of a proforma. Data management and statistical analysis were done using Statistical Package for Social Sciences (SPSS™) software, version 20. Point estimate at 95% Confidence Interval was calculated along with frequency and proportion for binary data.

## RESULTS

Out of 395 SN and NAP masses, 134 (33.92%) 29.26 - 38.58 at 95% Confidence Interval) were neoplastic lesions. The mean age of patients with neoplastic lesions was 48.54±21.31 years (49.14±21.43 years for male) and 47.83±21.48 years for female) ranging from 25 days to 92 years. Males comprised of 78 (58.2%) cases, and the females were 56 (41.7%) with a male to female ratio of 1.39:1.

Out of all neoplastic lesions, 60 (44.77%) were malignant, 73 (54.47%) were benign, and only one (0.74%) of the cases was intermediate. Distribution of benign and malignant lesions according to the genderand age group are tabulated below (Table 1, 2).

**Table 1:** Distribution of neoplastic lesions according to gender (n = 134

Type of Lesion	Gender		n (%)
	Female n (%)	Male n (%)	
Benign	27 (20.1)	46 (34.3)	73 (54.4)
Intermediate	1 (0.7)	0 (0.0)	1 (0.7)
Malignant^*^	28 (20.8)	32 (23.8)	60 (44.7)
Total	56 (41.7)	78 (58.2)	134 (100)

* Includes dysplastic lesions.

**Table 2:** Distribution of neoplastic lesions according to the age group (n=134).

Age Group	Type of Lesion			n (%)
	Benign n (%)	Intermediate n (%)	Malignant^*^ n (%)	
<10 Years	3 (2.2)	0 (0.0)	2 (1.4)	5 (3.7)
11-20 Years	11 (8.2)	0 (0.0)	1 (0.7)	12 (8.9)
21-30 Years	15 (11.1)	0 (0.0)	0 (0.0)	15 (11.1)
31-40 Years	10 (7.4)	1 (0.7)	4 (2.98)	15 (11.1)
41-50 Years	10 (7.4)	0 (0.0)	7 (5.2)	17 (12.6)
51-60 Years	9 (6.7)	0 (0.0)	19 (14.1)	28 (20.8)
61-70 Years	9 (6.7)	0 (0.0)	13 (9.7)	22 (16.4)
>70 Years	6 (4.4)	0 (0.0)	14 (10.4)	20 (14.9)
Total	73 (54.4)	1 (0.7)	60 (44.7)	134 (100.0)

* Includes dysplastic lesions.

Among 134 cases, nasal cavity (NC) was the most common site of biopsy comprising 107 (79.8%) of cases, followed by NAP 18 (13.4%), and Paranasal Sinus (PNS) 4 (2.9%). NC was also the commonest site for benign neoplasms comprising 65 (48.5%) cases. Only one lesion of intermediate malignancy in the series also occurred in the NC. Distribution of lesions according to the site is given in the table below (Table 3).

**Table 3:** Distribution of lesions according to the site (n=134).

Lesion	Nasal Cavity (NC) n (%)	Nasopharynx (NAP) n (%)	Paranasal Sinus (PNS) n (%)	NC+NP n (%)	NC+PNS n (%)	NC+NP+PNS n (%)	Total n (%)
**Benign**	65 (48.5)	3 (2.2)	2 (1.4)	0	3 (2.2)	0	73 (54.4)
**Intermediate**	1 (0.7)	0	0	0	0	0	1 (0.7)
**Malignant** ^*^	41 (30.5)	15 (11.1)	2 (1.4)	0	1 (0.7)	1 (0.7)	60 (44.7)
**Total**	107 (79.8)	18 (13.4)	4 (2.9)	0	4 (2.9)	1 (0.7)	134 (100)

* Includes dysplastic lesions.

In our study, inverted papilloma was the most common benign lesion comprising 28 (20.8%) of total lesions, whereas squamous cell carcinoma (SCC) and nasopharyngeal carcinoma (NPC) were the most common malignant lesions comprising 12 (8.9%) cases each.

All the neoplastic lesions of the study are depicted. Only one (0.7%) intermediate lesion occurred which was hemangioendothelioma (Table 4).

**Table 4:** Neoplastic lesions of the study (n = 134).

Lesion	n (%)	Lesion	n (%)
**Benign Lesions**	73 (54.4)		
Inverted Papilloma	28 (20.8)	Meningioma	1 (0.7)
Capillary Hemangioma	23 (17.1)	Nevus	1 (0.7)
Angiofibroma	8 (5.9)	Nasal Glioma	1 (0.7)
Squamous Papilloma	7 (5.2)	Sebaceous Adenoma	1 (0.7)
Cavernous Hemangioma	2 (1.4)	Trichoepithelioma	1 (0.7)
**Intermediate Lesions**	1 (0.7)		
Epithelioid Hemangioendothelioma			1 (0.7)
**Malignant Lesions**	60 (44.7)		
Squamous Cell Carcinoma	12 (8.9)	Olfactory Neuroblastoma	2 (1.4)
Nasopharyngeal Carcinoma	12 (8.9)	Positive for Malignancy	2 (1.4)
Basal Cell Carcinoma	6 (4.47)	Rhabdomyosarcoma	2 (1.4)
Sinonasal Undifferentiated Carcinoma	5 (3.7)	Sinonasal Adenocarcinoma	2 (1.4)
Non-Hodgkin Lymphoma	5 (3.7)	Malignant Peripheral Nerve Sheath Tumor	1 (0.7)
Adenoid Cystic Carcinoma	3 (2.2)	Osteosarcoma	1 (0.7)
Malignant Melanoma	3 (2.2)	Poorly Differentiated Carcinoma	1 (0.7)
Inverted Papilloma with Dysplasia	2 (1.4)	Nasal Polyp with Dysplasia	1 (0.7)

## DISCUSSION

Nasal cavity (NC), paranasal sinuses (PNS) and nasopharynx (NAP) harbors a variety of neoplastic lesions.^4,9,10^ In the present study, there was a male preponderance with a male to female ratio of 1.39:1. Other similar studies also showed a male predominance.^4,9,10^ In contrast, study of Parajuli S. et al. done in the central region of the country, and that of Bakari A. et al. from Nigeria showed female predominance.^2,11^

Benign lesions were most prevalent in the study comprising 54.47% of cases, whereas 44.7% of the cases were malignant. Majority of the studies also showed benign lesions as the most prevailing lesions, and the prevalence of malignant lesions ranged from 2.6% to as high as 43.8%.^2,5,9-13^ Neoplastic lesion occurring in the minimum age of our study (25 days) was found to be a benign lesion viz. capillary hemangioma.

Age-wise prevalence showed a fairly uniform distribution of lesions from the 2nd decade till the 7th decade, after which there is a decline in the incidence. The malignant lesions are seen increasing only after the 6th decade. Other studies also show a comparable observation and showed increase in prevalence of malignant lesions only after the 5th decade of life.^3,10,11,14^

Inverted papilloma (INP) was the most prevalent benign neoplastic lesion in the current study comprising 20.89% of all benign lesions. Studies done by Bakari A, et al. and Garg, et al. showed comparable observations.^2,9^ Different to our finding, hemangioma was the most common benign neoplasm in the study of Lathi A. et al, and capillary hemangioma in the study of Parajuli S, et al.^10,11^ INPs are characterized by local invasion, tendency for recurrence, and their association with malignancy.^15^ Study done by Krouse JH found squamous cell carcinoma (SCC) associated with INP in 9.1% of the cases, whereas Weissler MC, et al. and Lawson W, et al. reported 4.72% and 5.74% cases of malignancy respectively.^16–18^ Two cases (1.4%) in our study showed dysplasia in INP. These lesions might serve as a precursor lesion for SCC.

Current study also observed 1 (0.7%) case each of meningioma and nasal glioma (NG). Extracranial meningiomas (ECM) are rare and found primarily in the head and neck region involving ear, nose and PNS.^19^ Primary ECMs comprise less than 2% of all meningiomas, and their pathogenesis is not completely established. Entrapment of meningocytes during closure of midline structures creating disconnection with the neuraxis and formation of ectopic site for arising ECMs is postulated.^20^ NGs also arise from abnormal embryonal development. Radiological investigation is essential to exclude extracranial extension.^21^

This study showed 60 (44.77%) malignant cases of neoplastic cases. Similar to our finding, Wang X, et al. observed 43.8% malignant cases in their study, whereas study of Parajuli S, et al. and Singh SG found only 6.7% and 9% malignant lesions respectively.^1,11,13^ The prevalence of malignant lesions varies according to the geographical regions along with ethnic and cultural differences. Malignant lesions arising in the sinonasal and nasopharyngeal region may be confused with nasal polyps or other chronic inflammatory lesion causing delay in the diagnosis and management.^9^

Most common malignancies in the current study were SCC and nasopharyngeal carcinoma (NPC) comprising 8.9% each of all the lesions. Study of Jaison J, et al. also revealed 6.73% SCC as the primary malignancy. However, their study didn't have a single case of NPC among 104 cases studied over a period of 5 years.^22^ Similarly, study of Raj JA, et al. also showed SCC as their most common malignancy.^8^ SN and NAP region comprise only 3-5% of all head and neck malignancies, and SCC is the most common subtype consisting more than 50% of cases.^23,24^ Although the pathogenesis of SCC is poorly understood, carcinogen exposure due to occupational hazards, viral oncogenesis related to Human Papilloma Virus, INP as a precursor lesion, and a state of ongoing chronic inflammation are related towards the causation of SCC in the sinonasal and nasopharyngeal region.^24,25^

Study of Begum MS, et. al confirmed NPC as the most common malignant lesion comprising 18.18% of the cases, whereas in the study of Khan N, et al. it was the second most common malignancy comprising 4.16% of all lesions.^26,27^ NPC is a malignancy with poor prognosis, and has a distinct geographical distribution and is mostly centered in east and southeast Asia. Dietary habits, lifestyle, and environmental exposures including role of Epstein Barr Virus (EBV) are the related factors.^28^ EBV DNA can be used for screening people in endemic areas, as well as in the prognostic implementation, predicting treatment response, and disease surveillance.^29^

Present study found that sinonasal undifferentiated carcinoma (SNUC) comprised 5 (3.7%) cases. Study of Khan N, et al. showed presence of 3.12% SNUC in their study, whereas study of Parajuli S, et al. didn't show a single case of SNUC.^11,26^ SNUC is a malignancy with a propensity to metastasize, and usually presents at advanced stage leading to usually a poor prognosis with higher rates of recurrence. Despite advances in different modalities of treatment, the survival remains poor.^30^

Non-Hodgkin lymphoma (NHL) also comprised 5 (3.7%) of all cases in the study. Almost similar to our findings, study of Boer CV, et al. showed 0.12% NHL, Khan N, et al. observed 2% NHL, and Parajuli S, et al. found the incidence of lymphoma to be 3% in their study.^11,26,31^ In contrast, Zhu L, et al. showed 21.7% incidence of NHL.^32^ NHL in the sinonasal and nasopharyngeal regions are uncommon and range from different B'cells and T'cells lymphomas to Natural Killer/T-cell lymphomas.^33^ Many studies have pointed out diffuse large B'cell Lymphoma as the most common lymphoma in these anatomic regions.^34-36^ Immunohistochemistry and flowcytometry are two essential diagnostic modalities to sub-classify NHL leading to targeted therapy. However, due to lack of these facility, only morphological classification using National Cancer Institute working formulation were reported.^37^

Salivary gland-type adenoid cystic carcinoma and primary nasal mucosal melanoma comprised 3 cases (2.23%) each. Other malignant lesions like olfactory neuroblastoma, rhabdomyosarcoma, and sinonasal adenocarcinoma comprised 2 cases (1.49%) each, whereas 1 cases each (0.74%) of malignant peripheral nerve sheath tumor and osteosarcoma were seen. One of the case (0.74%) was signed out as poorly differentiated carcinoma, as it could not be further subclassified on histopathology. These malignant lesions are seen in a more or less similar fashion of incidence, or were absent throughout many studies.^2,3,5,10,11,26^

Although these malignant lesions are not common, identification in histopathology is crucial for their proper management.

## CONCLUSIONS

The sinonasal and nasopharyngeal anatomic region encompassed different neoplastic lesions in our study. Inverted papilloma was the most common benign lesion whereas both squamous cell carcinoma and nasopharyngeal carcinoma constituted the predominant malignant lesions. Histopathologic analysis of biopsy specimens is key to their final definitive diagnosis leading to their early and appropriate management, especially in the cases of malignant lesions. Screening can also be carried out for presence of any masses in older adults or elderly people.

## References

[1] Singh SG, Qureshi S, Jain L, Jadia S, Sharma S (2018). Presentation of Lesions of Nose and Paranasal Sinuses at a Tertiary Care Center in Central India.. Indian J Otolaryngol Head Neck Surg..

[2] Bakari A, Afolabi OA, Adoga AA, Kodiya AM, Ahmad BM (2010). Clinico-pathological profile of sinonasal masses: an experience in national ear care center Kaduna, Nigeria.. BMC Res Notes.

[3] Ajiya A, Abdullahi H, Shuaibu IY (2020). Clinicopathologic profile of sinonasal neoplasia in Kano, Northwestern Nigeria: A 10-year single-institution experience.. Ann Afr Med..

[4] Zafar U, Khan N, Afroz N, Hasan SA (2008). Clinicopathological study of non-neoplastic lesions of nasal cavity and paranasal sinuses.. Indian J Pathol Microbiol..

[5] Bist SS, Varshney S, Baunthiyal V, Bhagat S, Kusum A (2012). Clinico-pathological profile of sinonasal masses: An experience in tertiary care hospital of Uttarakhand.. Natl J Maxillofac Surg..

[6] Tan BK, Kern RC, Schleimer RP, Schwartz BS (2013). Chronic Rhinosinusitis: The Unrecognized Epidemic.. Am J Respir Crit Care Med..

[7] Modh SK, Delwadia KN, Gonsai RN (2013). Histopathological spectrum of sinonasal masses-A study of 162 cases.. Int J Cur Res Rev..

[8] Raj JA, Sharmila PS, Shrivastava M, Mahantachar V, Rajaram T (2013). Morphological spectrum of lesions in the sinonasal region.. J Evol Med Dent Sci..

[9] Garg D, Mathur K (2014). Clinico-pathological Study of Space Occupying Lesions of Nasal Cavity, Paranasal Sinuses and Nasopharynx.. J Clin Diagn Res..

[10] Lathi A, Syed MM, Kalakoti P, Qutub D, Kishve SP (2011). Clinico-pathological profile of sinonasal masses: a study from a tertiary care hospital of India.. Acta Otorhinolaryngol Ital..

[11] Parajuli S, Tuladhar A (2013). Histomorphological spectrum of masses of the nasal cavity, paranasal sinuses and nasopharynx.. J Pathol Nepal..

[12] Humayun AHP, Huq AZ, Ahmed ST, Kamal MS, Bhattacharjee N (2010). Clinicopathological study of sinonasal masses.. Bangladesh J Otorhinolaryngol..

[13] Wang X, Shi G, Liu Y, Ji H, He M, Li J (2011). [Analysis of the clinical and pathological characteristics of sinonasal neoplasms].. Lin Chuang Er Bi Yan Hou Tou Jing Wai Ke Za Zhi J Clin Otorhinolaryngol Head Neck Surg..

[14] Poursadegh M, Poursadegh F, Esmaeili M, Bakhshaee M (2015). Epidemiological Survey of Sinonasal Malignancy in North-East Iran.. Iran J Otorhinolaryngol..

[15] Melroy CT, Senior BA (2006). Benign sinonasal neoplasms: a focus on inverting papilloma.. Otolaryngol Clin North Am..

[16] Lawson W, Benger J, Som P, Bernard PJ, Biller HF (1989). Inverted papilloma: an analysis of 87 cases.. The Laryngoscope..

[17] Krouse JH (2001). Endoscopic treatment of inverted papilloma: safety and efficacy.. Am J Otolaryngol..

[18] Weissler MC, Montgomery WW, Turner PA, Montgomery SK, Joseph MP (1986). Inverted papilloma.. Ann Otol Rhinol Laryngol..

[19] Landini G, Kitano M (1992). Meningioma of the mandible.. Cancer..

[20] Aiyer RG, Prashanth V, Ambani K, Bhat VS, Soni GB (2013). Primary Extracranial Meningioma of Paranasal Sinuses.. Indian J Otolaryngol Head Neck Surg..

[21] Ma KH, Cheung KL (2006). Nasal glioma.. Hong Kong Med J..

[22] Jaison J, Tekwani DT (2015). Histopathological lesions of nasal cavity, paranasal sinuses and nasopharynx.. Ann Appl Bio-Sci..

[23] Ansa B, Goodman M, Ward K, Kono SA, Owonikoko TK, Higgins K (2013). Paranasal sinus squamous cell carcinoma incidence and survival based on Surveillance, Epidemiology, and End Results data, 1973 to 2009.. Cancer..

[24] Elgart K, Faden DL (2020). Sinonasal Squamous Cell Carcinoma: Etiology, Pathogenesis, and the Role of Human Papilloma Virus.. Curr Otorhinolaryngol Rep..

[25] Lewis JS (2016). Sinonasal Squamous Cell Carcinoma: A Review with Emphasis on Emerging Histologic Subtypes and the Role of Human Papillomavirus.. Head Neck Pathol..

[26] Khan N, Zafar U, Afroz N, Ahmad SS, Hasan SA (2006). Masses of nasal cavity, paranasal sinuses and nasopharynx: A clinicopathological study.. Indian J Otolaryngol Head Neck Surg..

[27] Begum MS, Sarker UK, Islam MA, Sangma MA, Paul P, Rahman MA (2018). Magnetic Resonance Imaging in Evaluation of Sinonasal Masses with Histopathological Correlation.. Mymensingh Med J..

[28] Lee HM, Okuda KS, González FE, Patel V (2019). Current Perspectives on Nasopharyngeal Carcinoma.. Adv Exp Med Biol..

[29] Chen Y-P, Chan ATC, Le Q-T, Blanchard P, Sun Y, Ma J (2019). Nasopharyngeal carcinoma.. Lancet Lond Engl..

[30] Abdelmeguid AS, Bell D, Hanna EY (2019). Sinonasal Undifferentiated Carcinoma.. Curr Oncol Rep..

[31] Van den Boer C, Brutel G, de Vries N (2010). Is routine histopathological examination of FESS material useful?. Eur Arch Otorhinolaryngol..

[32] Zhu L, Fang P, Liu Y, Tong B (2015). Clinical and diagnosis analysis of malignant lymphoma in nasal cavity and paranasal sinus.. Lin Chuang Er Bi Yan Hou Tou Jing Wai Ke Za Zhi..

[33] Hussein MR (2018). Non-Hodgkin's lymphoma of the oral cavity and maxillofacial region: a pathologist viewpoint.. Expert Rev Hematol..

[34] Steele TO, Buniel MC, Mace JC, El Rassi E, Smith TL (2016). Lymphoma of the nasal cavity and paranasal sinuses: A case series.. Am J Rhinol Allergy..

[35] Peng KA, Kita AE, Suh JD, Bhuta SM, Wang MB (2014). Sinonasal lymphoma: case series and review of the literature.. Int Forum Allergy Rhinol..

[36] Hsueh C-Y, Yang C-F, Gau J-P, Kuan EC, Ho C-Y, Chiou T-J (2019). Nasopharyngeal Lymphoma: A 22-Year Review of 35 Cases.. J Clin Med..

[37] Swerdlow SH, Cook JR (2020). As the world turns, evolving lymphoma classifications-past, present and future.. Hum Pathol..

